# Effects of Pravastatin on Type 1 Diabetic Rat Heart with or without Blood Glycemic Control

**DOI:** 10.1155/2018/1067853

**Published:** 2018-02-28

**Authors:** Jeong Jin Min, Byung-Seop Shin, Jong-Hwan Lee, Yunseok Jeon, Dae Kyun Ryu, Sojin Kim, Young Hee Shin

**Affiliations:** ^1^Department of Anesthesiology and Pain Medicine, Samsung Medical Center, Sungkyunkwan University School of Medicine, Seoul, Republic of Korea; ^2^Department of Anesthesiology and Pain Medicine, Seoul National University Hospital, Seoul National University College of Medicine, Seoul, Republic of Korea

## Abstract

Although statins have been suggested to attenuate the progression of diabetic cardiomyopathy, its effect without glycemic control remains unclear. Therefore, we evaluated the effect of pravastatin on diabetic rat hearts according to glycemic control. Rats were randomly divided into five groups: control (C), diabetes (D), diabetes with insulin (I), diabetes with pravastatin (P), and diabetes with insulin and pravastatin (IP). Eight weeks after allocated treatments, the heart was extracted and analyzed following echocardiography. Cardiac fibrosis was measured using Masson's trichrome stain. Cardiac expression of collagen I/III, matrix metalloproteinase (MMP)-2, MMP-9, and angiotensin-converting enzyme (ACE)/ACE2 was evaluated by immunohistochemistry and/or Western blot. Enzyme-linked immunosorbent assay was used for measuring reactive oxygen species (ROS). Diabetic groups without glycemic control (D and P) showed significantly impaired diastolic function and increased levels of cardiac fibrosis, collagen I/III, MMP-2, MMP-9, and ROS production. However, there were little significant differences in the outcomes among the control and two glucose-controlled diabetic groups (I and IP). Groups C and IP showed more preserved ACE2 and lower ACE expressions than the other groups did (D, I, and P). Our study suggested glycemic control would be more important to attenuate the progression of diabetic cardiomyopathy than pravastatin medication.

## 1. Introduction

Statin therapy is generally recommended in diabetic patients with cardiovascular risk factors or overt cardiovascular disease [1]. Many previous studies have shown that statins are effective to prevent major cardiac and cerebrovascular events [2, 3], which are related to the so-called “pleotropic” actions of statins including anti-inflammation, antithrombotic, antiatherosclerosis, antiproliferation, and reducing oxidative stress [4].

Statin treatment has also been suggested to reduce the progression of diabetic cardiomyopathy. Diabetic cardiomyopathy, which was first identified in 1972, is a separate disease from other well-established diabetic cardiovascular complications (e.g., coronary artery disease and cardiac autonomic neuropathy) and increases the risk of heart failure and cardiac mortality [5, 6]. Protective effects of statins on diabetic cardiomyopathy have been reported to improve cardiovascular remodeling by suppression of arterial wall thickening and myocardial fibrosis, restoration of left ventricular diastolic function, preservation of angiotensin-converting enzyme-2 (ACE2), and reduction of oxidative stress [7–10].

However, when using lipophilic statins (i.e., atorvastatin, simvastatin, or rosuvastatin) in diabetic patients, concerns regarding the progression of glucose intolerance [11] through alteration of insulin sensitivity or glucose uptake by adipocytes remain [12–16]. In contrast to those lipophilic statins, hydrophilic statins such as pravastatin and fluvastatin do not deteriorate glycemic control [7, 11, 14–16] because of the pharmacokinetic differences including hydrophilicity [4, 17].

In a previous study, we suggested that a combination of fluvastatin and insulin appears to be more effective than insulin alone in the diabetic heart [8]. However, considering that glucose control by insulin is the main therapy for diabetes, whether a clinical dose of statin would be protective in cases where blood glucose is not controlled remains unclear. In addition, the protective effects of statins on the diabetic heart may differ among statins. Therefore, in the present study, we evaluated the effect of pravastatin on cardiac function, myocardial fibrosis, oxidative stress, and ACE2 expression according to the presence of glycemic control in diabetic rat hearts. We hypothesized that early cotreatment of pravastatin and insulin would have more beneficial effects on cardiac protection than insulin-alone treatment.

## 2. Materials and Methods

This study was reviewed and approved by the Animal Care and Use Committee at Samsung Medical Center (IACUC number 20140203003), and the animal experiments were conducted in accordance with the Samsung Biomedical Research Institute guidelines for animal experiments.

### 2.1. Experimental Animal Modeling and Care

Seven-week-old male Lewis (inbred Wistar) rats (*n* = 87) were used in this study. All rats were housed at 22 ± 3°C with 40–60% humidity in a 12/12-hour light/dark cycle. All animal experiments were performed in a semipathogen-free barrier zone at the Samsung Laboratory Animal Research Center. Rats were randomly allocated into one of 5 groups according to the insulin or pravastatin treatment after diabetic modeling: (i) group C (control rats, *n* = 16), (ii) group D (untreated diabetic rats, *n* = 18), (iii) group I (diabetic rats receiving insulin treatment, *n* = 18), (iv) group P (diabetic rats receiving pravastatin treatment, *n* = 17), and (v) group IP (diabetic rats receiving cotreatment of insulin and pravastatin, *n* = 18). Diabetes was induced by an intravenous injection of 65 mg/kg streptozotocin (STZ); in group C, the same volume of citrate buffer (pH 4.5) was administered. Three days after STZ injection, blood glucose levels were measured using a OneTouch Ultra® Blood Glucose Meter (LifeScan Inc., Milpitas, CA), and a glucose level higher than 300 mg/dl was considered as a successful diabetic induction. In the groups treated with insulin (group I and group IP), the blood glucose level of each rat was measured twice daily (at 7 a.m. and 7 p.m.) and Lantus® (insulin glargine, Sanofi-Aventis, France) was administered at 2–10 U/kg to achieve a normal blood glucose range for eight weeks from the day of diagnosis. In the statin treatment group (group P and group IP), a calculated dose of pravastatin (20–25 mg/kg/day) was dissolved in water and orally administered once every evening by an oral gavage using a feeding catheter for eight weeks from the day of diagnosis. Standard rat chow and tap water were provided *ad libitum* throughout the study period.

### 2.2. Echocardiography

Eight weeks after treatment, each group of rats was anesthetized with isoflurane/oxygen, and transthoracic echocardiographic images were acquired using a Vevo® 2100 high-resolution imaging system (VisualSonics Inc., Toronto, Canada). Two-dimensional parasternal short-axis images were measured at the level of the midpapillary muscle, and M-mode tracings were recorded. Left ventricular end-diastolic area, left ventricular end-systolic area, left ventricular internal dimension in diastole, and left ventricular internal dimension in systole were measured, and left ventricular fractional area change (LV-FAC) and left ventricular fractional shortening (LV-FS) were calculated as previously described [8]. Mitral peak flow velocities at early diastole and atrial contraction were recorded using a pulsed Doppler technique in parasternal long-axis view, and the ratio of the early (E) to late (A) ventricular filling velocities (E/A ratio) was calculated. All measurements were performed by an experienced technician who was blinded to animal group allocation. Three representative cardiac cycles were analyzed and averaged for each measurement.

### 2.3. Biochemical Measurements

After echocardiography, blood glucose levels and body weights were measured. Blood samples were also collected from the abdominal aorta and then immediately centrifuged to measure total cholesterol, low-density lipoprotein, high-density lipoprotein, and triglycerides. Euthanasia was performed by cervical dislocation under deep anesthesia, and the heart was extracted and weighed.

### 2.4. Histology and Immunohistochemistry

Hearts extracted from 9–11 rats were formalin fixed, paraffin embedded, and sliced into 4 *μ*m sections. Sections were deparaffinized in xylene, rehydrated in graded alcohol, and transferred to 0.01 M phosphate-buffered saline (PBS, pH 7.4). Myocardial collagen depositions were stained by Masson's trichrome and measured. Conventional immunohistochemistry for collagen I/III, angiotensin-converting enzyme (ACE), and ACE2 expressions was performed by the same method as previously described [8]. For analyses, slides were scanned using a ScanScope AT digital slide scanner (Aperio, Vista, CA), and images were analyzed using the Aperio ImageScope Positive Pixel Count algorithm. To quantify immunohistochemical expression, 7 to 10 fields in each slide (at a magnification of 20 times) were randomly chosen and color positivity of pixels within the fields was automatically detected after setting the appropriate input parameters. Color positivity was presented as three different levels, strong positive, moderate positive, and weak positive, according to the strength of the color expression. For further analyses, we defined positivity as follows:

(1) $$\begin{array}{c} Positivity=\frac{number\text{ of }\left(strong\;positive+moderate\;positive\right)\;pixels}{total\;number\text{ of }pixels\text{ in the }selected\;fields}. \end{array}$$

### 2.5. Western Blot Analysis

Proteins were extracted from heart tissue obtained from 9–11 rats for each experimental group. Cardiac myocytes were placed on ice for 15 minutes and then sonicated for 30 s; whole-cell protein lysates were recovered by centrifugation. The protein concentration of the supernatant was determined using a Bradford reagent method (Bio-Rad, Hercules, CA, USA). Proteins were resolved by electrophoresis by sodium dodecyl sulfate-polyacrylamide gel electrophoresis (SDS-PAGE) or native PAGE as appropriate under denaturing conditions. The proteins were transferred to nitrocellulose membranes (BioTrace™ NT, Pall Corp., USA). After blocking in Tris-buffered saline with Tween 20 (TBS-T, 10 mM Tris, 150 mM NaCl, 0.1% Tween 20) containing 5% bovine serum albumin (BSA) for one hour, the membranes were incubated overnight with primary antibody in 3% BSA in TBS-T at 4°C. Membranes were then washed with TBS-T and incubated in 3% BSA in HRP-conjugated IgG secondary antibodies for 1 hour. Membranes were washed again with TBS-T, and the immunocomplexes were detected using a chemiluminescence reagent kit (Amersham Corp., Arlington Heights, IL, USA). The primary antibodies used for immunoblotting studies were collagen I antibody (ab6308, Abcam Inc.), collagen III antibody (ab7778, Abcam Inc.), matrix metalloproteinase- (MMP-) 2 antibody (ab79271, Abcam Inc.), MMP-9 antibody (ab137867, Abcam Inc.), ACE antibody (ab11734, Abcam Inc.), or ACE2 antibody (ab87436, Abcam Inc.). Immunoblotting for *β*-actin was performed to verify equivalent protein loading.

### 2.6. Enzyme-Linked Immunosorbent Assay (ELISA)

The concentration of reactive oxygen species (ROS) in the rat myocardium was measured using a commercial rat ROS ELISA kit (CSB-E15037r, CUSABIO®, Wuhan, Hubei, China). In brief, rat heart tissue was mechanically homogenized in PBS and stored overnight at −20°C. After two freeze-thaw cycles to break the cell membranes, the homogenized samples were centrifuged for 5 min at 5000 rpm and 4°C. Each supernatant was then transferred into a fresh test-tube and stored at −80°C, and the level of ROS was determined by ELISA kit following the manufacturer's recommended procedures.

### 2.7. Statistical Analysis

All statistical analyses were performed using SPSS for Windows (version 21.0, SPSS Inc., Chicago, IL, USA). Data are presented as median (interquartile range, IQR). Normality of the data distribution was determined with a Shapiro-Wilk test. Differences among groups were tested using a one-way analysis of variance or a Kruskal-Wallis one-way analysis of variance on ranks as appropriate, followed by Tukey's test using ranks for multiple comparisons. In Western blot analysis, the protein expression result in group C was used as a reference (with a reference value of “1”), and a one-sample signed rank test with Bonferroni correction was used when comparing group C with other groups (groups D, I, P, and IP). A *P* value less than 0.05 was considered statistically significant.

## 3. Results

### 3.1. Demographic and Laboratory Characteristics (Lipid Profile)

Diabetes was successfully induced in all rats. Of the 87 rats used in the experiment, 3 rats expired (2 died during diabetic modeling, and 1 from group D died after the echocardiographic exam), and thus 84 rats were included in the final analysis.  Table 1 shows the demographic and laboratory data of the rats after 8 weeks of each treatment. Diabetic rats without glycemic control (group D and group P) showed significantly lower body and heart weights compared with the other groups. For the lipid profile, group D showed the highest blood triglyceride level. However, group P, which was the diabetic group receiving pravastatin single treatment, showed a comparable lipid profile results to groups I and IP. The insulin-treated diabetic group without statin therapy (group I) showed comparable levels of triglycerides and low-density lipoproteins with rats on statin therapy (group P and group IP).

### 3.2. Echocardiography (Cardiac Function)

The echocardiographic data after 8 weeks of treatment are shown in Table 1. The median values of LV-FAC in diabetic rats without glycemic control (group D and group P) were significantly reduced compared with those in the other groups. The values of LV-FS were also lower in group D and group P compared with those in group C and group IP. The E/A ratio, which indicates left ventricular diastolic function, was significantly lower in group D and group P than in the other groups (Table 1).

### 3.3. Histology

**Figure 1:**
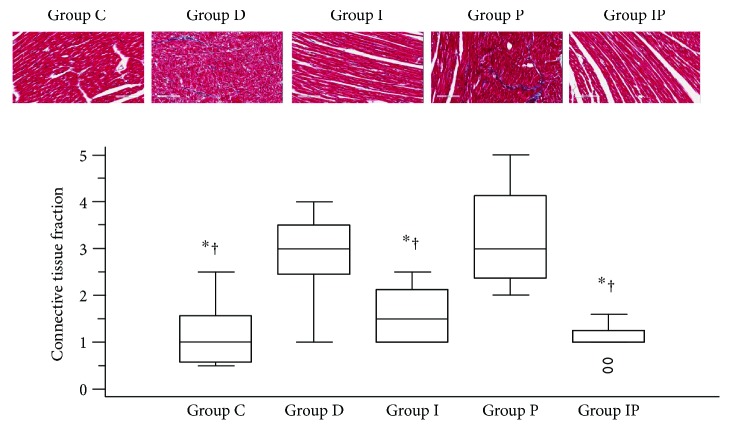
Masson's trichrome stain of the myocardium with the connective tissues stained blue. Boxes and lines represent medians and interquartile ranges, respectively. Dots represent the outliers. The upper part represents the images (with 20 times magnification) and the lower part shows the quantitative analysis. ^∗^*P* < 0.05 versus group D, ^†^*P* < 0.05 versus group P.

Masson's trichrome was used to stain the cardiac connective tissue blue. There were significant differences in the median cardiac connective tissue fraction among the five groups (*P* < 0.0001). The cardiac connective tissue fraction was significantly higher in the diabetic rats with poor glycemic control (group D and group P) than other groups (Figure 1). However, there were no significant differences in the cardiac connective tissue fraction among groups C, I, and IP (Figure 1).

### 3.4. Collagen and MMP-2, MMP-9 Protein Cardiac Expression

**Figure 2:**
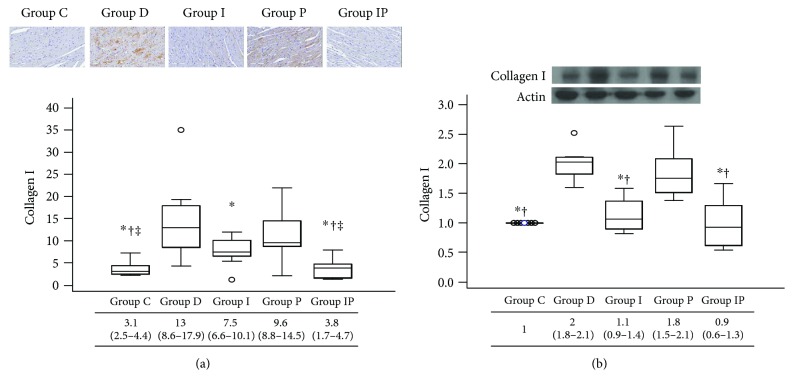
Representative micrograph of immunohistochemistry (a) and Western blot photograph for cardiac protein expression (b) of collagen I. Boxes and lines represent medians and interquartile ranges, respectively. Dots represent the outliers. The upper part represents the images (with 20 times magnification in immunohistochemistry) and the lower part shows the quantitative analysis. ^∗^*P* < 0.05 versus group D, ^†^*P* < 0.05 versus group P, and ^‡^*P* < 0.05 versus group I.

**Figure 3:**
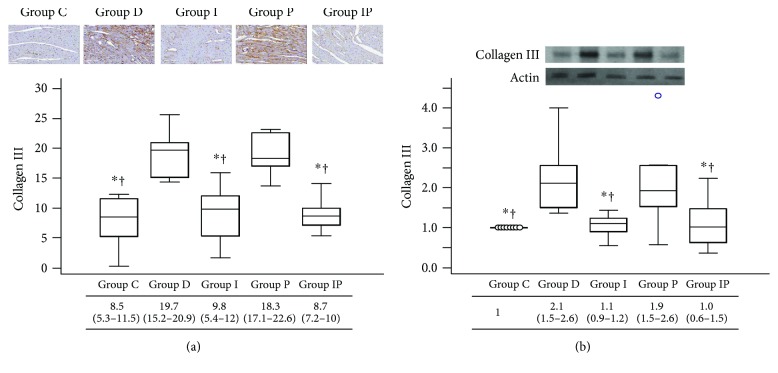
Representative micrograph of immunohistochemistry (a) and Western blot photograph for cardiac protein expression (b) of collagen III. Boxes and lines represent medians and interquartile ranges, respectively. Dots represent the outliers. The upper part represents the images (with 20 times magnification in immunohistochemistry) and the lower part shows the quantitative analysis. ^∗^*P* < 0.05 versus group D, ^†^*P* < 0.05 versus group P.

Immunohistochemistry and Western blot analyses demonstrated that cardiac expression of collagen I and III was significantly increased in group D and group P compared with those in the other groups (Figures 2 and 3). Group I showed higher expression of collagen I by immunohistochemistry analysis compared with group C and group IP (Figure 2(a)). However, expression of collagen III shown by immunohistochemistry (Figure 3(a)) and collagens I and III shown by Western blot analyses results (Figures 2(b) and 3(b)) was comparable among group C, group I, and group IP.

**Figure 4:**
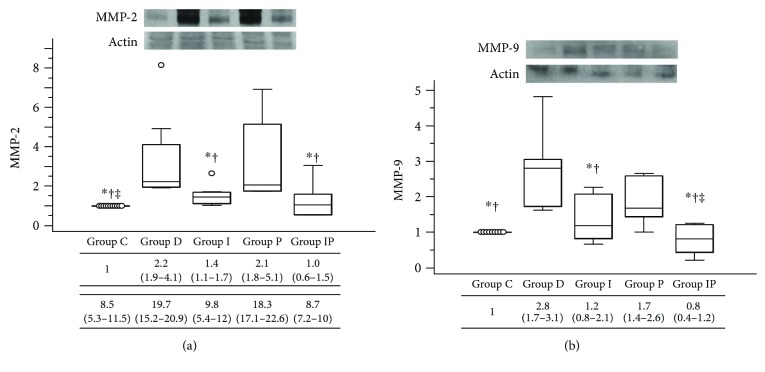
Representative Western blot photographs for cardiac protein expression of MMP-2 (a) and MMP-9 (b). Boxes and lines represent medians and interquartile ranges, respectively. Dots represent the outliers. ^∗^*P* < 0.05 versus group D, ^†^*P* < 0.05 versus group P, and ^‡^*P* < 0.05 versus group I. MMP: matrix metalloproteinase.

Cardiac expression of MMP-2 and MMP-9 was significantly increased in the high blood glucose groups (group D and group P) as compared with those in other groups as shown by Western blot analyses (Figure 4). The MMP-2 expression in group I was higher than group C, which was comparable to the expression level of group IP (Figure 4(a)). Group C and group IP showed comparable results in both MMP-2 and MMP-9 expression levels (Figures 4(a) and 4(b)).

### 3.5. ROS Production

**Figure 5:**
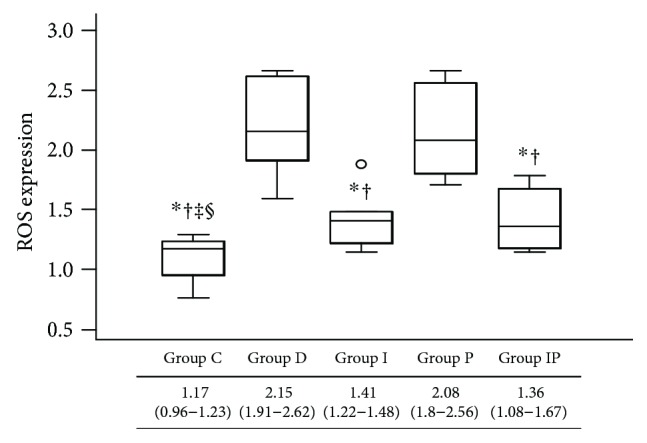
Cardiac expressions of reactive oxygen species measured by enzyme-linked immunosorbent assay. Boxes and lines represent medians and interquartile ranges, respectively. Dots represent the outliers. ^∗^*P* < 0.05 versus group D, ^†^*P* < 0.05 versus group P, ^‡^*P* < 0.05 versus group I, and ^§^*P* < 0.05 versus group IP.

The ELISA demonstrated that the median reactivity of ROS in heart tissue homogenate was significantly increased in group D and group P compared with the other groups (Figure 5). Group C showed the lowest value of ROS reactivity. No significant differences in ROS reactivity were observed between group I and group IP (Figure 5).

### 3.6. Cardiac ACE and ACE2 Protein Expression

**Figure 6:**
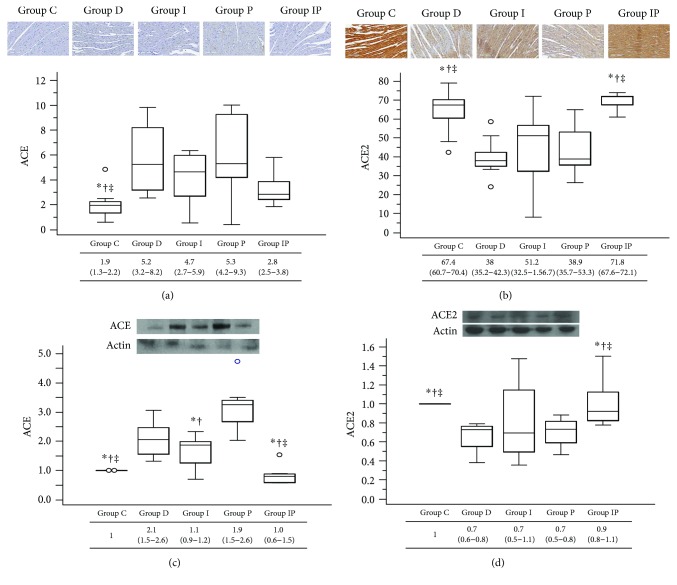
Representative micrographs of immunohistochemistry for ACE (a) and ACE2 (b) and Western blot photographs for cardiac protein expression for ACE (c) and ACE2 (d). Boxes and lines represent medians and interquartile ranges, respectively. Dots represent the outliers. The upper part represents the images (with 20 times magnification in immunohistochemistry) and the lower part shows the quantitative analysis. ^∗^*P* < 0.05 versus group D, ^†^*P* < 0.05 versus group P, and ^‡^*P* < 0.05 versus group I. ACE: angiotensin-converting enzyme.

In immunohistochemistry analysis, the ACE expression level was significantly lower in group C compared to groups D, I, and P, while ACE expression in group IP showed no significant difference from that of the group C (Figure 6(a)). For ACE2, higher expressions were observed in groups C and IP compared to the other groups (Figure 6(b)). In Western blot analyses, ACE protein expression was significantly lower in groups C and IP than in the other groups (Figure 6(c)). In contrast, ACE2 cardiac protein expression was significantly preserved in groups C and IP compared to those in the other groups (Figure 6(d)).

## 4. Discussion

### 4.1. Major Findings

In this experimental study, we observed the cardiac effects of pravastatin under two different conditions of blood glucose control in the STZ-induced diabetic rat model. Under poor glycemic control, diabetic rats showed significantly impaired cardiac function and increased levels of cardiac fibrosis and ROS production regardless of pravastatin treatment. When blood glucose levels were well controlled from the early stage of diabetes with insulin, diabetic rats showed comparable results with nondiabetic rats in cardiac function, fibrosis, MMP expression, and ROS production, regardless of additional treatment with oral pravastatin. Although cardiac ACE2 expression seemed to be more preserved in the combination treatment group receiving pravastatin and insulin than in the insulin-alone group, this did not lead to meaningful differences in other outcomes in our study.

### 4.2. Pathophysiology of Diabetic Cardiomyopathy and Statin

Diabetic cardiomyopathy is defined as left ventricular dysfunction in diabetic patients without coronary artery disease or other potential etiological conditions [18] and, morphologically, is mainly characterized by cardiac hypertrophy and adverse remodeling associated with myocardial fibrosis. The pathogenesis of diabetic cardiomyopathy has a multifactorial basis. Several suggested mechanisms include metabolic disturbances, insulin resistance, microvascular disease, renin-angiotensin system activation, and excessive oxidative stress [5]. Among the various mechanisms, the role of oxidative stress has been strongly suggested in many studies [19–22], and therefore beneficial effects of statins and antioxidant treatments on the management of diabetic complications have been extensively investigated and reported.

### 4.3. Results of Our Study

Previously, we suggested that a combination of fluvastatin and insulin may be more effective than insulin-alone treatment in diabetic hearts with regard to reducing myocardial fibrosis and preserving cardiac function and ACE2 expression [8]. However, in that study, the sole effect of fluvastatin on diabetic cardiomyopathy could not be explained because of the absence of an experimental group receiving a single fluvastatin treatment. In the present study, we included experimental groups observing both the sole effect of each treatment (group I or group P) and the effect in combination (group IP) on diabetic rat hearts. Our results suggest that glycemic control is the primary factor involved in reducing the progression of diabetic cardiomyopathy. Contrary to our expectations, once blood glucose was well controlled with insulin, the addition of pravastatin treatment did not make a significant difference in most of the study outcomes. Two insulin-treated groups with good glycemic control (group I versus group IP) showed comparable results regardless of the use of pravastatin in cardiac function, fibrosis, collagen expression, MMP activation, or ROS production.

Moreover, between the two diabetic groups without glycemic control (group D versus group P), pravastatin treatment did not bring any beneficial effects compared to the untreated group showing comparable progression of diabetic cardiomyopathy. These results are inconsistent with previous findings that statin treatment itself was effective in reducing myocardial fibrosis and cardiac dysfunction in the diabetic condition without specific comments for glycemic control [7].

There are several explanations for the results of our study. First, because we started and maintained good glycemic control from a very early stage of diabetes, diabetic complications may have been prevented or delayed in the insulin-treated rats during our study period. In clinical practice, a chronic hyperglycemic period generally exists until the initial diagnosis of diabetes, and in this situation, increased levels of inflammatory proteins and oxidative stress are not completely restored even with the later normalization of blood glucose [23]. This phenomenon results from “glycemic memory,” in that once ROS production is increased in the early hyperglycemic environment, ROS-induced cellular dysfunction and mitochondrial DNA damage may occur and persist independently with later glycemic control. Secondly, the different dosage of pravastatin used for treatment in our study (20–25 mg/kg/day) may have affected our results. In previous experimental studies, much higher doses of pravastatin (100 mg/kg/day) effectively reduced cardiac fibrosis and MMP activity [24]. Another pravastatin study, which showed the preventive effect of cardiovascular remodeling in a type II diabetic model, also used higher dose of pravastatin (100 mg/kg/day) [7]. However, considering that the recommended dosage of pravastatin is 40 mg/day in human clinical practice, the pravastatin dosage we used was not an underdose for rats of much smaller size than human. Third, the results may be due to the unique pharmacokinetic characteristics of pravastatin such as lower plasma protein binding, hydrophilic nature, different primary metabolic pathways, and dual routes of elimination [17, 25, 26]. Pravastatin has shown different effects on the progression of glucose intolerance [11, 12] and side effects compared with other statins [27]. Moreover, in a previous study, which observed the effects of various statins for MMP activity and vascular smooth muscle proliferation, only pravastatin showed different results from other statins (fluvastatin, simvastatin, lovastatin, and atorvastatin) [28].

### 4.4. Statin and ACE2

Interestingly, cardiac ACE2 expression was more preserved in the pravastatin-treated group among glucose-controlled rats in our study. However, this did not lead to a meaningful difference in the levels of cardiac fibrosis or ROS production. The effects of pravastatin might not have been high enough to sufficiently reduce the MMP upregulation or cardiac collagen deposition. In our previous study, additive treatment with fluvastatin in glucose-controlled diabetic rats led to more preserved cardiac ACE2 expression as well as in the cardiac fibrosis and diastolic cardiac function than did the insulin-only treated group. Although fluvastatin is less potent than pravastatin with regard to lipid-lowering effects, the differences in pleiotropic effects among statins remain unclear because there are insufficient data.

### 4.5. Potential Benefits of Hydrophilic Statins in Diabetic Patients

When making a therapeutic selection between statin agents in diabetic patients for primary prevention purpose, there are some factors that must be considered. Although all statins are generally well tolerated, some side effects such as drug interactions, muscle toxicity, insomnia, and cognitive dysfunction have been related to statin use. These side effects are more likely to occur with the use of lipophilic statins. In this regard, hydrophilic statins have some advantages compared to lipophilic statins especially in diabetic or elderly patients taking multiple medications. Among hydrophilic statins, pravastatin has not deteriorated glycemic control and is less likely to displace albumin-bound drugs such as warfarin because of its lower plasma protein binding [26]. Moreover, in clinical situations where patients must receive multiple medications, pravastatin may have the fewest drug interactions because it is not metabolized by CYP450 [17]. In patients with impaired renal function, fluvastatin is preferred because fluvastatin is the drug that is least affected by renal elimination [17]. However, further evaluation is needed to identify appropriate agents and adequate doses to provide optimal therapeutic efficacy including the pleiotropic effect of statins for each individual patient's condition.

### 4.6. Study Limitations

There were several limitations in our study. First, since we started glycemic control immediately after the confirmation of diabetic induction, there was no experimental group with a chronic hyperglycemic period before starting insulin treatment, and we did not observe the effect of statin under such condition with glycemic memory, which is common in the real-life clinical situation. Second, we did not observe the long-term effects of pravastatin after 8 weeks. Although 8 weeks is sufficient to develop diabetic cardiomyopathy in the groups without glycemic control, a longer follow-up might be needed in groups with controlled-blood glucose because the onset and progression of diabetic cardiomyopathy may be delayed under good glycemic control. Moreover, lipid profiles were normalized in the pravastatin-treated groups, and therefore long-standing use of statins may make a difference even between the groups with poor glucose control because dyslipidemia may also play a role in the development of microvascular complications [5, 29, 30]. Third, we used semiquantitative methods to analyze the study outcomes (immunostaining, Western blotting, and ELISA), although they are popular techniques used in biomedical research. Fourth, our experimental model was type I diabetes induced by STZ injection. Although the STZ-induced diabetic rat model is an established experimental model for research of diabetic cardiomyopathy [8, 9, 31, 32] and models of type 1 and type 2 diabetes have been used interchangeably to understand pathophysiological mechanisms of diabetic cardiomyopathy [32], it is difficult to extend our study results to all diabetic conditions. Finally, the lipid-lowering effect of statin would be different in rodents; therefore, rat models might not be perfect to evaluate the effects of statin. However, in this experimental study, we aimed to evaluate the pleiotropic effects of statin independent from its lipid-lowering effects. Moreover, many previous studies have used rats or mice to evaluate the protective effects of statin on the cardiovascular system.

## 5. Conclusions

In conclusion, this experimental study with type 1 diabetic rat models revealed that glycemic control is the primary condition attenuating the progression of diabetic cardiomyopathy even with pravastatin treatment. Further studies may be needed to identify a regimen for optimal pleiotropic effects of pravastatin on the heart in different types of diabetes.

## Figures and Tables

**Table 1:** Demographic, laboratory, and echocardiographic data after eight weeks of treatment.

	Group C	Group D	Group I	Group P	Group IP	*P* value
BWt (g)	387 (375.8–401)^†, ‡, §, ‖^	226 (215–293)	352 (320–370)^†, §^	246.5 (236–255)	352 (329.5–366)^†, §^	<0.001
HWt (g)	1.18 (1.15–1.27)^†, ‡, §^	0.81 (0.77–0.85)^∗^^, ‡, ‖^	1.07 (0.98–1.11)^∗^^, †, §^	0.87 (0.80–0.92)^∗^^, ‡, ‖^	1.09 (1.02–1.17)^†, §^	<0.001
HWt/BWt (%)	0.31 (0.3–0.33)^†, §^	0.39 (0.36–0.41)^∗^^, ‡, ‖^	0.31 (0.3–0.34)^†, §^	0.35 (0.33–0.39)^∗^^, ‡^	0.32 (0.31–0.37)^†^	0.002
Blood glucose (mg/dl)	142 (133–153)^†, §^	443 (403–470)^∗^^, ‡, ‖^	141 (89–149)^†, §^	438 (417–505)^∗^^, ‡, ‖^	119 (83–151)^†, §^	<0.001
Lipid profile (mg/dl)						
Total cholesterol	82.5 (77–91.5)	93 (85.25–103)	81.5 (72–86)	79.5 (71–81)	88 (84–90)	0.01
Triglyceride	65 (41–77.5)^†^	145 (138–325.5)^∗^	124 (86–140)^∗†^	92.5 (74–137)^∗†^	106 (65–145.5)^∗†^	<0.001
HDL	74 (71.5–76.5)^†, ‡^	60 (58–74)^∗, ‖^	68 (60–72)^∗, ‖^	69 (65–75)^‖^	79 (74–81)^†, ‡, ‖^	0.004
LDL	11.5 (11-12.5)	15 (10–17.25)	11 (10–13.25)	10 (10-11)	12 (10.25–13)	0.118
Echocardiographic variables						
FAC (%)	61 (58–68)^†, §^	38 (29–43)^∗, ‡, §, ‖^	60 (53–62)^†^	52 (45–56)^∗, †, ‖^	63 (60–72)^†, §^	<0.001
FS (%)	53 (49–54)^†, ‡, §^	39 (37–43)^∗, ‖^	45 (41–48)^∗^	41 (34–45)^∗, ‖^	53 (41–63)^†, §^	0.0014
E/A ratio	1.8 (1.5–2.5)^†, §^	0.9 (0.8–0.9)^∗, ‡, ‖^	1.8 (1.2–1.9)^†, §^	0.8 (0.7–0.8)^∗, ‡, ‖^	1.7 (1.5–2.3)^†, §^	<0.001

Data are presented as the median (IQR). *P* values were calculated using a Kruskal-Wallis one-way analysis of variance on ranks. The Tukey test was used for multiple comparisons. BWt: body weight; HWt: heart weight; HDL: high-density lipoprotein; LDL: low-density lipoprotein; FAC: fractional area change; FS: fractional shortening; E/A: the ratio of the early (E) to late (A) ventricular filling velocities. ^∗^*P* < 0.05 versus group C, ^†^*P* < 0.05 versus group D, ^‡^*P* < 0.05 versus group I, ^§^*P* < 0.05 versus group P, and ^‖^*P* < 0.05 versus group IP.
